# The comparison of STA-MCA bypass and BMT for symptomatic internal carotid artery occlusion disease: a systematic review and meta-analysis of long-term outcome

**DOI:** 10.1186/s41016-021-00236-2

**Published:** 2021-04-05

**Authors:** Shifei Cai, Hao Fan, Chao Peng, Yuzhang Wu, Xinyu Yang

**Affiliations:** 1 Department of Neurosurgery, Tianjin Medical University General Hospital, Tianjin, 300052 China; 2 Department of Neuroophthalmology, Tianjin Medical University Eye Hospital, Tianjin, 300052 China

**Keywords:** STA-MCA bypass, BMT, Internal carotid artery occlusion, stroke

## Abstract

**Background:**

Superficial temporal artery (STA)-middle cerebral artery (MCA) bypass surgery is now being widely used in moyamoya disease, and its therapeutic value in SICAO remains divergent.

**Methods:**

A systematic search was performed in PubMed, EMBASE, and Cochrane Databases in Feb. 2020 and updated in Jun. 2019. We have strict inclusion and exclusion criteria. Cochrane Bias Risk Assessment Tool was used to assess the quality of included RCTs. Review Manager 5.3 was used for analysis results in terms of comparing the STA-MCA bypass and BMT. For dichotomous variable outcomes, risk ratios (RRs) and 95% confidence intervals (95%CIs) were calculated for the assessment.

**Results:**

The total patient cohort consisted of 2419 patients, of whom 1188 (49.1%) patients had been grouped in STA-MCA bypass and 1231 (50.9%) patients had been divided into the BMT group. Mean follow-up of included patients was 29 months. The RR of the seven studies was 1.01, and the 95% confidence interval was .89–1.15, with statistical significance, *Z* = .13, *P* = .89, sustaining that STA-MCA bypass was not superior to BMT in symptomatic carotid artery occlusion disease.

**Conclusions:**

STA-MCA bypass and BMT were associated with similar rates of a composite of long-term stroke. And the risk of long-term overall stroke was mildly higher with BMT. At present, each patient should receive more precise treatment, by reasonably assessing the individual differences of each patient to reduce the recurrence rate of stroke.

## Background

Superficial temporal artery (STA)-middle cerebral artery (MCA) bypass surgery is now being widely used in moyamoya disease, and its therapeutic value in symptomatic internal carotid artery occlusion (ICAO) remains divergent.

With a prevalence of less than 10%, carotid artery stenosis was regarded as a relatively rare disease among patients [1]. Unilateral ICAO is found in approximately 3% of the asymptomatic elderly population, and as the chief culprit of transient ischemic attacks (TIA), it was leading to more than 10% of TIA, as well as 15–25% of ischemic strokes [2]. Before bypass surgery, the common and effective treatment was best medical therapy (BMT), as time goes on, strides were aimed at perfecting STA-MCA bypass techniques to revascularize the circulation [3, 4]. In 1977, IEIBS (International Extracranial (EC)-Intracranial(IC) Bypass Study), an international multicenter randomized controlled study, supported by the National Institutes of Health (NIH), proved that STA-MCA bypass cannot effectively pull down the recurrence rate of ischemic stroke [5]. Around the same time, the Ministry of Health of Japan funded a multicenter randomized controlled study JET (Japanese EC-IC Bypass Trial) to test the academic hypothesis that recent symptomatic hemodynamic cerebral ischemia could significantly reduce after STA-MCA bypass in combination with BMT. Recently published meta-analysis [6] investigating treatment efficacy in patients with internal carotid artery near occlusion manifested that BMT alone is not superior to surgical (CEA or CAS) with respect to 30-day or 1-year stroke or death prevention. And Ogawa [7] holds that STA-MCA arterial bypass is beneficial for patients with symptomatic hemodynamic cerebral ischemia due to occlusive disease.

The aim of the context was to integrate the results of the randomized controlled trial throughout history to determine the optimal surgical strategy of any stroke or death within 2 years for ICAO.

## Methods

The study strictly adhering to the PRISMA statement [8] was approved by all collaborating authors of the Carotid Artery Occlusion Treatment Group and designed by the core study team.

### Search strategy and study eligibility

A systematic search of literatures, between Jan. 1985 and Jun. 2019, was performed in PubMed, EMBASE, and Cochrane Databases in Feb. 2020. “Carotid Artery, Internal”, “occlusion”, and “randomized controlled trial” were used to identify all relevant articles by subject word and free word search, which is integrally shown in Table 1. Two researchers screened the literature eligibility independently based on title and abstract, and disagreements were resolved by discussion with the senior author.

**Table 1:** Retrieval strategy for PubMed

Search	Query
#8	Search (#6 and #7)
#7	Search randomized controlled trial [Title/Abstract] OR controlled clinical trial [Title/Abstract]) OR randomized [Title/Abstract] OR randomly [Title/Abstract] OR Case-Control Studies [Title/Abstract] OR case control study [Title/Abstract]
#6	Search (#4 and #5)
#5	Search occlusion [Title/Abstract] OR occlusive [Title/Abstract]
#4	Search (#2 or #3)
#3	Search Internal Carotid [Title/Abstract] OR Artery, Internal Carotid [Title/Abstract] OR Carotid Arteries, Internal [Title/Abstract] OR Internal Carotid Arteries [Title/Abstract] OR Internal Carotid Artery [Title/Abstract]
#2	Search “Carotid Artery, Internal”[Mesh]

Studies were eligible if they reported on the following: all accepted articles were randomized controlled trials (RCT); subjects conformed to the criteria used to diagnose atherosclerotic internal carotid artery occlusion (AICAO); intervening measure of all studies must be extracranial-intracranial (EC-IC) bypass while the comparison measure was best medical therapy (BMT); the primary endpoint was all stroke or death from randomization within 2 years or longer; a minimum of ten patients with AICAO due to atherosclerosis; excluded were study type not explained, the data of outcomes, cannot acquire the full-text, animal studies, reviews, too small a sample size, and articles in languages other than English.

The first author of the original document was contacted by sending an email for no full-text included literature. If there was no response, the other authors of the paper were contacted similarly, a maximum of three attempts by two other authors.

### Study quality assessment

Cochrane Bias Risk Assessment Tool [9] for randomized controlled trial was used to assess the quality of included studies. Three researchers independently completed the literature quality evaluation according to Cochrane Bias Risk Assessment Tool, which mainly evaluates the risk of bias in six aspects, the select (including random sequence and allocation concealment), implementation of researchers and subjects (including blind), measuring result evaluation method for the blind (study), the follow-up data integrity (end), report (selective reports the results of the study), and other (bias source). Ambiguity is assessed by the senior author, if there was no coherence.

### Data extraction and study outcomes

After quality assessment and data examination, data was extracted from the original literature and analyses in this meta-analysis were based on randomized controlled trial.

Study, patient, and outcome characteristics were collected by two co-authors. Patient characteristics comprised the following: patient follow-up time, intervening measure (STA-MCA bypass or BMT), and the number of any stroke or death within 2 years or longer. Study characteristics comprised the following: year of study publication, number of included patients, and study type.

The primary outcome of the present study was any stroke or death within 2 years or longer. All the content of reports has been taken into account in our study.

### Ethical approval statement

All literature study was conducted based on published studies. Therefore, ethical approval or patient consent was available.

### Statistical analysis

Software Review Manager 5.3 was used for analysis results in terms of comparing the STA-MCA bypass and BMT. For dichotomous variable outcomes, risk ratio (RRs) and 95% confidence intervals (95%CIs) were calculated for the assessment. The data were considered to be heterogeneous when *I*^2^ > 50%; therefore, a meta-analysis was conducted by a random effects model according to the Cochrane Handbook for Systematic Reviews of Interventions (version 5.1.0). Otherwise, the fixed effect model was performed.

## Results

### Study eligibility

**Fig. 1:**
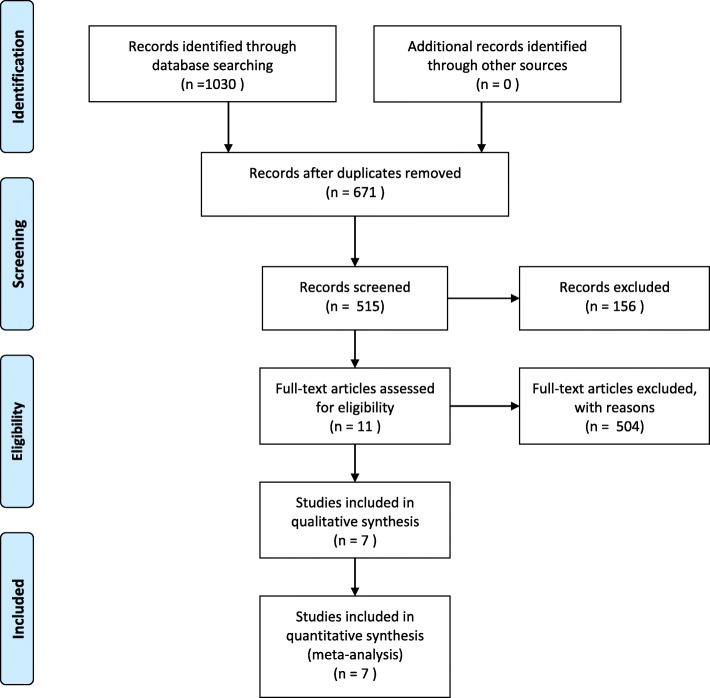
Flow diagram of document selection

The search yielded 1030 articles through database searching, of which 7 studies [2, 5, 7, 10–13] (Table 2) were included for quantitative synthesis (meta-analysis). In the process of literature selection, 156 of records were excluded with reasons of literature reviews, systematic reviews, reviews, animal experiments, etc., and 504 with reasons of literature with inconsistent study content or inconsistent intervention/control measures by reading the abstract. The process of selecting literature and the data extraction form are shown respectively in Fig. 1 and Table 3.

**Table 2:** Overview of included studies

Author	Year	Mean follow-up (months)	Patients (***n***)	Interventions (***n***)		Any stroke or death within 2 years
				STA-MCA bypass	BMT	
Tanahashi et al.	1985	29	60	38	22	42
IEIBS	1985	55.8	1377	663	714	413
Powers et al.	2011	24	195	97	98	40
Ogawa et al.	2012	24	206	103	103	24
Grubb et al.	2013	20 ± 3	190	93	98	6
Ma et al.	2016	24	195	97	98	42
Nahab et al.	2019	24	195	97	98	42

*STA-MCA bypass* Superficial temporal artery-middle cerebral artery bypass, *BMT* Best medical therapy, *RCT* Randomized controlled trial, *NA* Not available, *IEIBS* an International multicenter randomized controlled study, IEIBS, funded by the US National Institutes of Health

**Table 3:** Data extraction of included literature

Author	Year	STA-MCA bypass (no.)		BMT (no.)	
		Events (%)	Total	Events (%)	Total
Tanahashi et al.	1985	26 (68.4)	38	16 (73.0)	22
IEIBS	1985	206	663	207	714
Powers et al.	2011	20 (20.6)	97	20 (20.4)	98
Ogawa et al.	2012	7 (6.8)	103	17 (16.5)	103
Grubb et al.	2013	20 (21.5)	93	22 (22.4)	98
Ma et al.	2016	20 (21)	97	22 (22.7)	98
Nahab et al.	2019	22 (22.7)	97	20 (20.4)	98

### Quality assessment

**Fig. 2:**
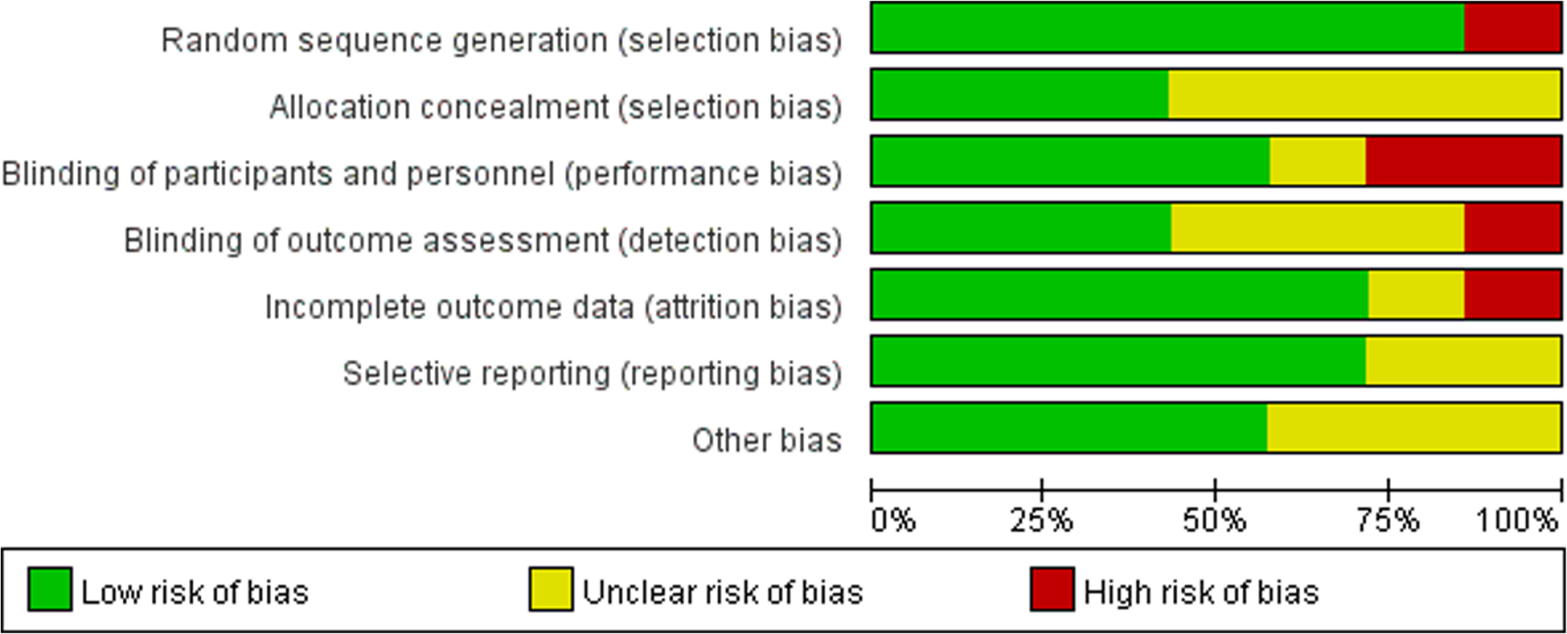
Risk of bias graph

**Fig. 3:**
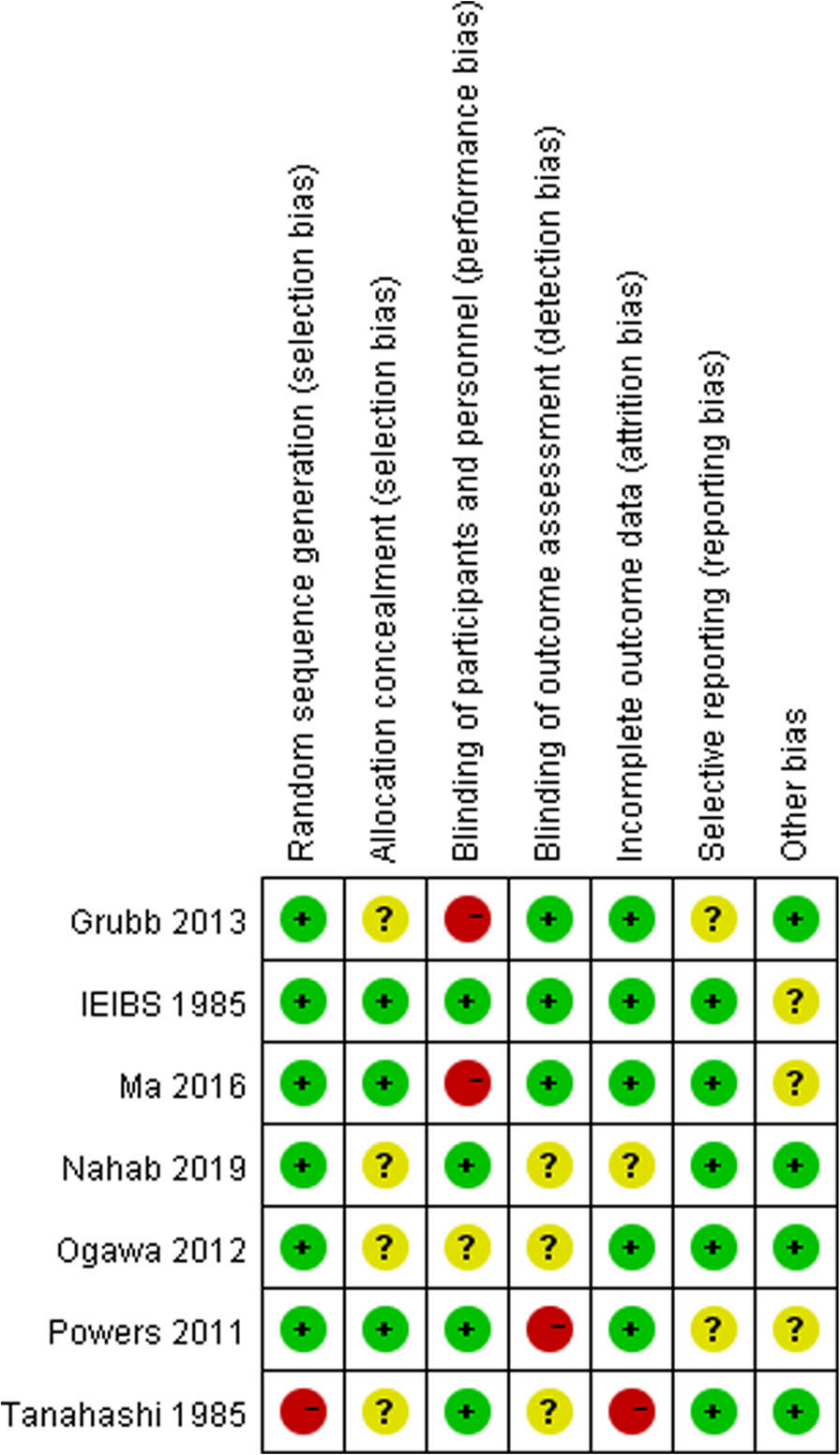
Risk of bias summary

The literature quality evaluation was conducted separately by three reviewers in terms of Cochrane Bias Risk Assessment Tool for randomized controlled trial. Detailed ratings could be available in Figs. 2 and 3.

### Study population

The total patient cohort consisted of 2419 patients, of whom 1188 (49.1%) patients had been grouped in STA-MCA bypass and 1231 (50.9%) patients had been divided into the BMT group. Mean follow-up of included patients was 29 months. Incidence rate of any stroke or death within 2 years or longer, respectively, was 70%, 20.5%, 11.6%, 3.2%, 21.5%, 30%, and 21.5%. In general condition, although many elements could influence the results, no significant discrepancy was found in this character of the two groups as shown in Table 4.

**Table 4:** Baseline characteristics of the included study

Author	Year	Age, mean (SD)		Bypass patency rates	Male, no. (%)		Diabetes mellitus, no. (%)		Previous stroke, no. (%)		Hypertension (%)	
		STA-MCA	BMT		STA-MCA	BMT	STA-MCA	BMT	STA-MCA	BMT	STA-MCA	BMT
Tanahashi et al.	1985	53.7 (9)	55.8 (10)	98%	31	14	STA-MCA group (26); BMT group (15)					
IEIBS	1985	56	56	96%	537 (81)	585 (82)	113 (17)	129 (18)	517 (78)	557 (78)	345 (52)	343 (48)
Powers et al.	2011	58 (9)	58 (9)	95%	69 (71)	61 (62)	21 (22)	23 (23)	44 (45)	35 (36)	76 (78)	77 (79)
Ogawa et al.	2012	63 (6)	60 (7)	98%	58 (56)	64 (62)	74 (71.8)	68 (66)	59 (57)	65 (63)	78 (74)	80 (77)
Grubb et al.	2013	61.1 (7.6)	57.8 (9.3)	97%	66 (71)	59 (60)	20 (22)	28 (29)	43 (46)	52 (53)	75 (81)	71 (72)
Ma et al.	2016	63	65	NA	48 (49.4)	52 (53)	NA	NA	NA	NA	NA	NA
Nahab et al.	2019	58.9 (7.6)	57.1 (9.6)	97%	31 (62)	35 (70)	10 (20)	14 (28)	33 (69)	27 (54)	40 (82)	43 (86)

### Any stroke or death within 2 years or longer

**Fig. 4:**
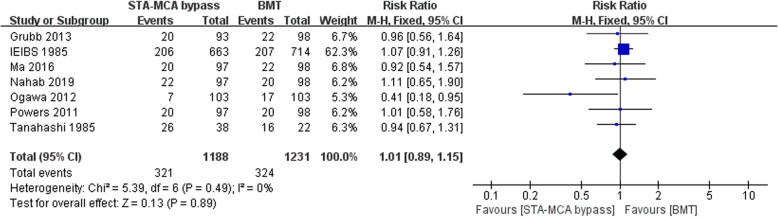
Forest plot of any stroke or death within 2 years or longer

**Fig. 5:**
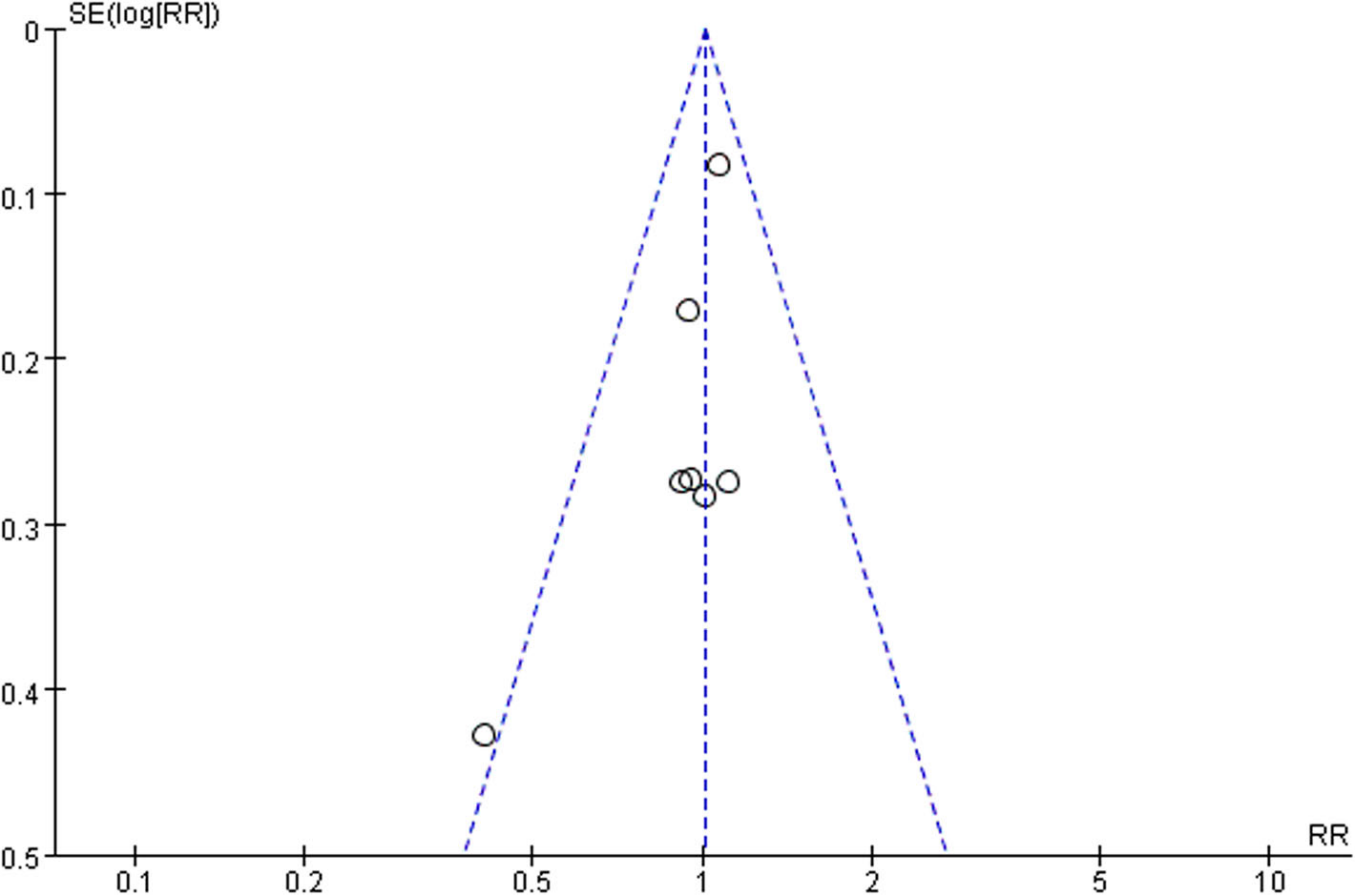
Funnel plot of publication bias

Seven articles contained the number of patients with postoperative stroke or death; there were 321 and 324 patients, respectively, that come out endpoint in the STA-MCA bypass and BMT groups, of which the long-term any stroke or death rate severally is 27.0% and 26.3%. According to *I*^2^ = 0% (< 50%) of the heterogeneity test, and *P* = .49 (> 0.1) of the *Q* test, it is demonstrated that the heterogeneity among the selected literatures has no statistical significance, and the fixed effect was selected for meta-analysis. The RR of the seven studies was 1.01, and the 95% confidence interval was .89–1.15, with statistical significance, *Z* = .85, *P* = .40 (> 0.05), sustaining that STA-MCA bypass was not superior to BMT in symptomatic carotid artery occlusion disease (Fig. 4). The funnel plot was used to investigate whether there was publication bias in this study, and the symmetry of the funnel plot meant that there was no publication bias (Fig. 5).

### Literature of recent 10 years

**Fig. 6:**
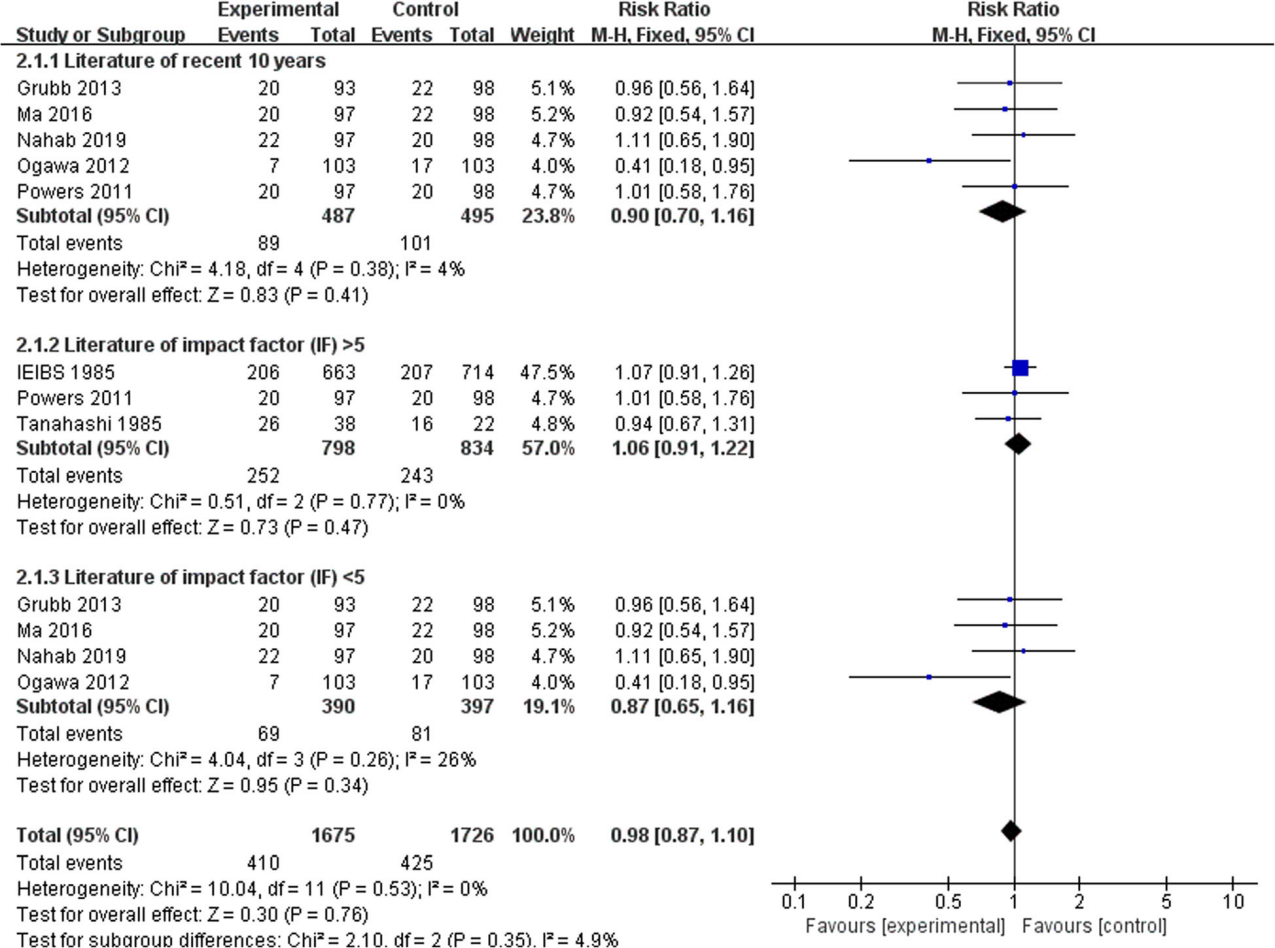
Forest plot of subgroup analysis

Five studies were totally included in this subgroup for analysis (Fig. 6). The meta-analysis revealed that there was no heterogeneity between STA-MCA bypass and BMT groups (*I*^2^ = 4%, *P* = .38). The fixed effect model was adopted to analyze and test for overall effect *Z* = .83 (*P* = .41) pointing out that STA-MCA bypass was not superior to BMT in symptomatic ICAO. All other statistical indicators were significant (RR = .90, 95%CI (.70–1.16)).

### Literature of impact factor (IF) > 5

Three studies were selected into this subgroup (Fig. 6). The meta-analysis indicated that no heterogeneity come under observation between STA-MCA bypass and BMT groups (*I*^2^ = 0%, *P* = .77). The fixed effect model was adopted to analyze and test for overall effect *Z* = .73 (*P* = .47) manifesting that there was no significant difference between STA-MCA bypass and BMT in symptomatic ICAO. All other statistical indicators were significant (RR = 1.06, 95%CI (.91–1.22)).

### Literature of impact factor (IF) < 5

Four studies were contained in this subgroup (Fig. 6). Indicating that heterogeneity existing between STA-MCA bypass and BMT groups (*I*^2^ = 26%, *P* = .26), the fixed effect model was adopted to analyze and test for overall effect (*Z* = .95 (*P* = .34)) manifesting that there was no significant difference between STA-MCA bypass and BMT in symptomatic ICAO. All other statistical indicators were significant (RR = .87, 95%CI (.65–1.16)).

### Bias test

Publication bias in this study was assessed with funnel plots investigating that the symmetry of the funnel plot meant that no evident publication bias (Fig. 5) was covered.

## Discussion

Our analysis, which included data from 7 RCTs and 2419 patients, demonstrated that the aggregate efficacy outcome of stroke during the non-periprocedural stroke did not differ significantly between STA-MCA bypass and BMT groups. ICAO stroke, the ravages of atherosclerosis [14, 15], accounts for 15% of all strokes, and the rate of ipsilateral stroke is 2.1~3.8% per year due to non-selective carotid artery occlusion in the USA. The mechanisms include downstream embolus production and residual embolism, among which cortical artery compensation after carotid artery occlusion may determine the recurrence of stroke [15]. Clinical symptoms of this kind of stroke are regularly associated with intracranial emboli in the distal carotid or middle cerebral arteries.

The extracranial section of the internal carotid artery occlusion (ICAO) was handled by surgical way for extracranial-intracranial vascular bypass, striding the lesion area, and improving distal vascular blood flow to reduce the risk of stroke and enhance local brain nerve function. In 1967, Yasargil performed the first procedure for a patient with middle cerebral artery (MCA) occlusion of Marfan’s syndrome. In 1985, Sundt et al. [16] retrospectively analyzed 415 cases of ischemic cerebrovascular patients undergoing STA-MCA bypass surgery in 8 years, showing the patency rate reached 99%, which was confirmed by digital subtraction angiography and transcranial Doppler. This series of retrospective studies strongly demonstrated the safety and efficacy of this procedure. This research continues to languish. However, considering that for a specific patient, Schmiedek et al. [17] thought ICAO does not always result in cerebral hemodynamic disorder due to the existence of compensatory mechanisms such as collateral circulation. It has become a key issue in relevant studies to evaluate the hemodynamic status of the patient and as one of the indications for intervention. In order to certify the above opinion, the team of Grubb et al. [18] conducted a prospective blind longitudinal cohort study, indicating that the incidence rate of all stroke in patients with oxygen extraction fraction (OEF) elevation was higher than that in patients with normal OEF, and the relative risk of all stroke and ipsilateral stroke caused by OEF elevation was 6.0 and 7.3, respectively, meaning that symptomatic ICAO of the extracranial segment is associated with a higher risk of subsequent ischemic stroke, particularly in patients with elevated OEF. For high-risk patients, extracranial-intracranial (EC-IC) bypass surgery could theoretically benefit from vascular bypass technology, since it reduces the percentage of OEF to normal levels.

When designing the Carotid Occlusion Surgery Study (COSS) study scheme, 40% of the incidence of stroke in the drug group was set according to previous studies, and the improvement of drug treatment resulted in a significant reduction of the incidence of stroke, resulting in the deviation of the original study scheme. COSS funded by the National Institutes of Health (NIH), showing 40% of the incidence of stroke in the drug group, was set according to previous studies. However, the improvement of drug treatment resulted in a significant reduction of the incidence of stroke, which led to the deviation of the original study scheme and the failure of COSS study. And this is the reason why we did not include this study in our discussion. Therefore, we conducted a subgroup analysis according to literature impact factor. In terms of relatively high-quality literature (IF> 5), there was no heterogeneity between STA-MCA bypass and BMT groups (*I*^2^ = 28%, *P* = .24). The random effect model was adopted to analyze and test for overall effect, *Z* = .29 (*P* = .77), manifesting that there was no significant difference between STA-MCA bypass and BMT in symptomatic ICAO. As for relatively poor-quality literature (IF < 5), heterogeneity existing between STA-MCA bypass, and BMT groups (*I*^2^ = 50%, *P* = .13), the random effect model was adopted to analyze and test for overall effect (*Z* = .72 (*P* = .47)) proving the same conclusion. In the aspect of the design of the test scheme of COSS, the patients with the highest potential risk of ischemia fail to be screened out due to the inclusion time of patients [19, 20] and the inclusion criteria of PET examination [21].

Some surgeons with 2-day training or less than 10 bypass surgeries were also admitted to the COSS, which may lead to an abnormal increase in the incidence of perioperative adverse events. To reduce the impact of surgical techniques on recurrent stroke and the effects of anesthesia, perioperative intensive care, and nursing strategies in our meta-analysis, we also carried out subgroup analysis with literature from the last decade. No obvious heterogeneity between the two groups (*I*^2^ = 28%, *P* = .24). Overall effect (*Z* = .61 (*P* = .54)) also pointed out that STA-MCA bypass was not superior to BMT in symptomatic ICAO.

Chronic hypoperfusion may generate plenty of adverse effects such as brain softening, decreased number of neurons, reduced brain volume, language impairment, and decreased cognitive function [22, 23]. A great part of previous clinical trials had focused only on severe stroke as the endpoint event but had failed to give equal weight to the life outcomes of long-term hypoperfusion or recurrent ischemic events. As far as our team is concerned, several possible reasons may be responsible for this phenomenon. First, although many clinical trials have been done using surgical techniques that were very sophisticated at the time, the technique of bypass surgery was still limited by the surgical capabilities of the surgeons and surgical facilities. Secondly, included clinical trials of EC-IC bypass did not distinguish end-to-end or end-to-side anastomosis. If end-to-side anastomosis is used, there is still a possibility of occlusion of the distal thrombus and abscission to the intracranial. Finally, previous studies did not analyze hemodynamic damage after bypass as an independent risk factor. As to whether such patients can benefit from bypass surgery, subsequent studies should not only devote to the recurrence rate of stroke in the short and long term, but also take other factors closely related to patients’ quality of life, such as cognitive function, as important indicators.

To sum up, our results are subject to the limitations inherent to meta-analyses involving the pooling of data from different trials with different study protocols, definitions of clinical outcomes, and baseline characteristics of patients. New multicenter randomized controlled studies will be conducted in evaluation of patients’ cerebral hemodynamic status, establishing accurate indicators of illness and efficacy as the endpoint and perfecting detailed inclusion criteria to improve the study protocol. Our meta-analysis has several advantageous features, including a greater number of patients and restriction to only large RCTs that are less likely to be subject to publication bias.

## Conclusions

STA-MCA bypass and BMT were associated with similar rates of a composite of long-term stroke. The risk of long-term overall stroke was mildly higher with BMT. At present, each patient should receive more precise treatment, by reasonably assessing the individual differences of each patient to reduce the recurrence rate of stroke.

## Data Availability

The datasets used and/or analyzed during the current study are available from the corresponding author on reasonable request.

## Abbreviations

STA-MCA bypass: Superficial temporal artery-middle cerebral artery bypass
ICAO: Internal carotid artery occlusion
TIA: Transient ischemic attacks
BMT: Best medical therapy
IEIBS: International Extracranial (EC)-Intracranial (IC) Bypass Study
NIH: National Institutes of Health
COSS: Carotid Occlusion Surgery Study
OEF: Oxygen extraction fraction
